# Pharmacists Becoming Physicians: For Better or Worse?

**DOI:** 10.3390/pharmacy6030071

**Published:** 2018-07-23

**Authors:** Eugene Y. H. Yeung

**Affiliations:** 1 Education Centre, Royal Lancaster Infirmary, Lancaster LA1 4RP, UK; eugeney@doctors.org.uk; Tel.: +44-1524-65944; 2 Department of Medical Microbiology, The Ottawa Hospital General Campus, The University of Ottawa, Ottawa, ON K1H 8L6, Canada

**Keywords:** collaboration, pharmacist, physician, pharmacy education, medical education, interprofessional, multidisciplinary, conflict, interview

## Abstract

Physicians and pharmacists nowadays are often described as adversaries rather than members of the same team. Some pharmacists apply to medical school later in their careers, and experience obstacles during the transition process. This article details interviews with two physician–pharmacists, who each have a past pharmacist license and current physician license. The respondents described the limitations of pharmacists’ scope of practice as their main reasons to pursue a medical career. However, the respondents enjoy applying their pharmacy knowledge and experience to improve their medical practice. They do not feel pharmacy seniors and medical recruiters are supportive towards their chase for medical careers. The respondents noted the importance of peer-reviewed articles to promote pharmacist involvement in patient care and collaboration between physicians and pharmacists. Conflicts between physicians and pharmacists tend to happen because of their different focuses on patient care. The respondents do not see themselves having an edge over other medical school applicants, and noted that recruiters could negatively view their pharmacy experience. The respondents believe that physician–pharmacists are catalysts to foster collaboration between physicians and pharmacists, because they clearly understand the role of each profession. Nevertheless, the respondents feel that physicians and pharmacists are generally lukewarm towards pharmacists transitioning into physicians.

## 1. Introduction

Prior to the Edict of Frederich II, which officially created the profession of pharmacy from medicine in the Western World, the roles of “prescriber” and “dispenser” were virtually identical [1]. One might expect pharmacy to inherit the cultural norms from medicine. However, physicians and pharmacists nowadays are often described as “adversaries rather than members of the same team” [2]. Some pharmacists apply to medical school later in their careers, and experience “culture shocks” during the process of becoming physicians [1]. The culture of “physician–pharmacists” (professionals with past pharmacist licenses and current physician licenses) is unique [1], but peer-reviewed literature in this area is very limited. The lack of literature could be due to pharmacists recognizing the adversarial relationship between pharmacy and medicine, and thus being secretive about their intention to pursue medical careers. Some may fear being accused of using pharmacy education as a stepping stone to medicine. For instance, the pharmacy director of Mount Sinai Hospital, Canada, questioned whether pharmacists are “MD wannabees” for performing physical assessment [3]. 

Perhaps the best people to comment on physician–pharmacist collaboration would be those who completed the undergraduate training and understand the hardship in both professions. The current commentary adapted the self-interview style of a published qualitative study [4], in which pharmacists shared how they integrated experience into practice, managed challenges, and advised for pharmacists wishing to do the same. The aim of the interview was to explore details about physician–pharmacists’ culture and suggestions on how to improve interprofessional collaboration between the two professions. As a disclaimer, the two physician–pharmacists’ responses do not represent the views of all pharmacists and physicians, and are meant to be starting points for future research.

## 2. Materials and Methods 

The semistructured interviews were undertaken with two ex-pharmacists who are now physicians, and follow the methods adapted from two previous studies [1,4]. As an inclusion criterion, each participant must have a past pharmacist license and current physician license. No specific exclusion criteria were used. The author administered the questions through key informant interviews, asked for elaboration of answers, and compiled and edited the manuscript. The interview consisted of open questions about the participants’ background, experience as pharmacists and physicians, motivation to study medicine, and their opinions on how to improve collaboration between pharmacy and medicine. All respondents provided informed consents and received no monetary compensation for their participation.

## 3. Results

Question 1: Describe your pharmacy experience and motivation to study medicine.

Response: I worked as a pharmacist in both community and hospital settings for years. I also completed a research degree in pharmacy to explore my scope of practice. However, I was not satisfied with my jobs at that time. For example, when working in community, I had no access to patients’ clinical and laboratory findings, and thus found it difficult to monitor patients’ drug effectiveness and adverse effects. When I asked physicians to provide patients’ clinical details and management plans, I did not always feel welcomed. Similarly, when I asked patients to provide more details about their illness, some questioned my intention and simply said their physicians alone are sufficient to manage their health. Not every pharmacy employer appreciates your research experience; some can see you as being a “non-clinical” person. These experiences make me want to take a more comprehensive role in health care and pursue a medical career.

Response: Before going to medical school, I worked as a community pharmacist, which I very much enjoyed. However, I also like hands-on experience with patients, which I experienced during my volunteer work in a labor and delivery unit in a developing country. I realized I could practice surgeries and clinical procedures as a physician, but probably not as much as a pharmacist. However, I still appreciate the training I received in pharmacy, which is applicable to my current medical practice, especially when managing patients with multiple morbidities.

Question 2: What have been some of the most satisfying experiences that you had using your pharmacy knowledge?

Response: I taught medical students on how to dose vancomycin and gentamicin, and apply pharmacokinetics in clinical settings, because medical students have limited exposure in these areas. I shared my clinical pharmacology knowledge to improve the antimicrobial stewardship program in a teaching hospital [5]. In other projects, I used my pharmacy experience to advocate for better medication reconciliation [6], adverse drug reaction documentations [7], and collaboration between physicians and pharmacists [8]. Pharmacists can improve public perception by publications and word of mouth, especially from physician–pharmacists. For example, I suggested modifications of pharmacy training to improve their physical examination skills [9]. Since becoming a physician, I still actively contribute to pharmacology research and have published the findings in medical journals [10,11,12].

Response: Pharmacology knowledge is especially useful in clinics, where we prescribe for patients with menopause, infertility, and abnormal uterine bleeding. It is also useful in gyne-oncology, antepartum, and postpartum wards, where we encounter various medications beyond the usual scope of practice of obstetricians and gynecologists. Although our specialty physicians are very familiar with medications commonly used in our field, we need additional help managing patients’ multiple morbidities—that is when general knowledge on medications could help. For example, I helped patients to differentiate some over-the-counter medications, and advised them to check with their community pharmacists. I feel I empower my pharmacy colleagues, and facilitate seamless care and interprofessional collaboration.

Question 3: What barriers have you faced when applying to medical schools?

Response: Not all pharmacy seniors are supportive. When I asked for reference letters, one replied, “If you want career counselling, my door is always open. I have seen this movie many times.” That comment did not discourage me, but motivated me to persevere in my application.

Response: Contrary to popular belief, I do not see obvious advantages of being a pharmacist. Some medical recruiters may think you are having a good life even without becoming a physician. Why should they not give the medical school positions to candidates who sacrifice all their lives to become physicians? Other candidates also have their own skill sets that make them favorable in medical school admissions.

Question 4: What are ongoing challenges in encouraging pharmacist involvement in medical settings?

Response: During a presentation, a medical colleague said research studies conducted by physicians are more clinically relevant than ones conducted by other healthcare professionals, which are sometimes self-glorifying their own practices. I cannot completely disagree—I often see articles about the benefit of pharmacy services in pharmacy journals; in contrast, physicians tend to be well-regarded in the public eye, and do not need as much validation of their services. There are peer-reviewed articles that question the benefits of pharmacists in multidisciplinary care [13,14], that led me to write letters to editors to express my concerns [15,16]. I understand that peer-reviewed articles can improve the image of the pharmacy profession, but believe the focus should now be shifted towards how collaboration with physicians improves patients’ clinical outcomes. However, when submitting articles about physician–pharmacist collaboration to pharmacy journals, my collaborators and I received responses like “pharmacists do not want to become physicians.” Journal reviewers and editors denied our topic being relevant to pharmacy practice, and questioned our insight and knowledge about pharmacy.

Response: It is not easy for pharmacists to meet all physicians’ expectations. Physicians’ clinics tend to run late and overtime [17,18], so they may not want pharmacists to interrupt for trivial issues. But some other physicians complain about pharmacists changing medications without informing physicians beforehand. Some physicians dislike the long waiting time in pharmacy and cost of dispensing fees. But many physicians’ clinics also have long waiting times and charge the government consultation fees. There are still prejudices towards pharmacists and double-standards on both professions.

Question 5: Why do you think conflicts happen between physicians and pharmacists?

Response: Physicians and pharmacists have different priorities. The most important drug-related problem is not always the most important medical issue. Hospital physicians, for example, tend to focus on patients’ main reasons for admissions, whereas pharmacists want to take care of all drug-related issues. Once, a pharmacist urged to restart a myocardial infarcted patient’s vitamin tablets; my medical colleagues questioned whether the situation was urgent. Another reason for conflicts is that physicians do not want to work beyond their usual competency level, as recommended by their regulatory bodies, but pharmacists tend to feel competent in any drug-related issues. For instance, a pharmacist asked a general pediatric staff physician to change a psychiatric medication started by a community psychiatrist; the pediatrician had to pass the issue to the patient’s general practitioner because that is out of her area of expertise; the general practitioner might have to hand over the issue to the psychiatrist, due to worries of changing the psychiatrist’s management plan. Physicians have prescribing power, but are not allowed to freely prescribe anything they want. When their suggestions are denied, pharmacists could interpret that as their service being undervalued.

Response: In pharmacy school, I was taught to actively look for drug-related problems and ask the prescribers to make changes. It seems like a sign of professional achievement when pharmacists successfully intervene in patients’ medical therapies. I heard of a pharmacist wanting to start a patient on metformin despite the patient’s poor prognosis after a cardiac arrest. On the contrary, physicians like to keep patients’ management plans status quo. Physicians’ goals of care are usually bringing a patient’s clinical status back to baseline, safely discharging the patient home, and monitoring in community settings. As the Hippocratic Oath states, first do no harm. Making unnecessary therapeutic changes could put a patient at a higher risk of experiencing adverse effects, and delay one’s discharge date. Another common area of conflicts is when to order an investigation. Many hospital pharmacists are knowledgeable about the tests available, and tend to promptly recommend a test to rule in a diagnosis. I heard of a pharmacist suggesting a d-dimer test to rule in thromboembolism in a septic shock patient. However, physicians are trained to ensure the clinical presentations and examination findings fit with the working diagnosis, and avoid ordering tests when the clinical situations show low predictive values [19]. Physicians are taught the art of reassurance to avoid unnecessary testing, which wastes healthcare funding and causes health anxiety for patients [20].

Question 6: What is your opinion on pharmacists wanting to become physicians?

Response: Physicians and pharmacists alike need to be open-minded. Yes, you lose a pharmacist to medical school, but then you have a physician who truly understands the benefits of physician–pharmacist collaboration to health care. As an ex-pharmacist, I tend to be interested in anything drug-related, including research and clinical work in any specialties. This curiosity could come back to haunt me. In an interview, I was criticized for lack of focus in one specialty, and questioned about my intent to apply for the job position. I jokingly rebutted, “Well, I played a lot of sports too. It does not mean I want to be a professional athlete.” None of interviewers even smiled. Apparently, we did not share the same sense of humor.

Response: Many physicians applied more than once to medical school. After years of medical school, one needs to complete years of postgraduate training. Family and financial factors play big roles in your decision-making—you should not become physicians simply because of the perceived stable income. You should be humble and not see yourselves as having an edge over other applicants.

## 4. Discussion

We identified similar perspectives of physician–pharmacists who integrate their pharmacy experience into medical practices. They have faced barriers in applying to medical schools, which stemmed from the adversarial relationship between pharmacy and medicine. They advocated collaboration between physicians and pharmacists, but are aware of its difficulty. A major barrier is the different priorities between physicians and pharmacists, which were also noted in a survey conducted on pharmacists and physicians [21]. The study showed that physicians want pharmacists to help with insurance approvals and medication counselling, whereas pharmacists want to manage patients’ drug-related problems.

One possible solution for interprofessional strain is collaborative teaching to medical and pharmacy students, which can be used as a bridge to understand each other’s role and minimize unnecessary conflicts in practice [8]. The University of British Columbia (Canada) has long developed elective interprofessional modules for students in various health disciplines [22]. However, studies on interprofessional education tend to show aversion towards teaching delivered by the other healthcare professional [5,23]. Interprofessional education requires further honing to improve physicians’ and pharmacists’ acceptance towards each other.

The respondents illustrated how their use of pharmacy knowledge results in more comprehensive care. They understand the limitation of their involvement if they were not physicians, and described the double standard on physicians and pharmacists. The findings are consistent with a previous qualitative study on physician–pharmacists, which described the physician–pharmacist relationship like the Canada–US (or New Zealand–Australia) effect: physicians are like Americans who were born through liberty and revolution, whereas pharmacists are like Canadians who compromise and negotiate [1]. Similarly, a semistructured interview with 11 pharmacists and 8 physicians concluded that pharmacists confer trust based on ones’ title, degree, and status, whereas physicians build trust based on ones’ competency and performance [24]. Like physicians and pharmacists, other professions also experience intra- and interprofessional strains on status and prestige [25].

The respondents described the importance of peer-reviewed publications to better define pharmacists’ roles and debunk myths. These perspectives illustrated how physician–pharmacists are catalysts to foster collaboration between physicians and pharmacists, because they clearly understand the role of each profession. Nevertheless, pharmacists may face obstacles in applying to medical schools, as both physicians and pharmacists are reportedly lukewarm towards how pharmacists transition into physicians.

The findings of the current article add to the limited peer-reviewed literature on the unique culture of physician–pharmacists [1]. Although there are online forums and Youtube videos on how pharmacists transition into physicians, these are not peer-reviewed and are thus omitted in the current article. Limitations of this article include a small sample size, which makes broad generalizations of the findings difficult. It is difficult to recruit licensed physicians who had pharmacist licenses and are willing to participate in an interview with no monetary compensation, and, thus, selection bias might have occurred. It is understandable that some interview questions, such as commenting on why conflicts happen between the professions, are controversial and involve critique on how each profession practices. That makes the recruitment process even more difficult. It is noted that the responses from the two physician–pharmacists could be opinions but not facts. The respondents do not represent all pharmacists, especially those who work in other countries and experience different educational systems. These limitations are common among qualitative studies, especially the ones on innovative ideas. Qualitative studies on how pharmacists perform physical examinations and contribute to general practice recruited only 3 and 16 respondents, respectively [4,26]. A hypothesis was generated in the current article that encourages researchers to conduct future research on the culture and struggles of physician–pharmacists. With more studies, we would collectively increase the sample size and make the findings more generalizable.

## 5. Conclusions

From the perspective of the two interview respondents, physician–pharmacists understand the culture of each profession, and are good liaisons to promote interprofessional collaboration. They believe they can integrate their pharmacy knowledge to aid their practice in medicine. Nevertheless, the respondents feel that physicians and pharmacists are generally lukewarm towards pharmacists transitioning into physicians. The current article has a sample size of only two physician–pharmacists, but their responses are good starting points for future research. We require more peer-reviewed articles and research to explore the unique culture of physician–pharmacists, and their contribution towards the healthcare system.

## References

[1] Austin Z., Gregory P.A.M., Martin J.C. (2007). Negotiation of interprofessional culture shock: The experiences of pharmacists who become physicians. J. Interprof. Care.

[2] Ma C.S.J., Holuby R.S., Bucci L.L. (2010). Physician and pharmacist collaboration: The University of Hawai’i at Hilo College of Pharmacy—JABSOM experience. Hawaii Med. J..

[3] Simpson S.H., Wilson B. (2007). Should pharmacists perform physical assessments?. Can. J. Hosp. Pharm..

[4] Chua D., Ladha F., Pammett R.T., Turgeon R.D. (2017). Pharmacist performance of physical assessment: Perspectives of clinical pharmacists working in different practice settings. Can. J. Hosp. Pharm..

[5] Yeung E.Y.H., Alexander M. (2017). Use of junior doctor-led peer education to improve antibiotic stewardship. Br. J. Clin. Pharmacol..

[6] Yeung E.Y.H. Are We Legitimately Stopping Medications? Use of Pharmacist and Junior Doctor Teaching to Improve Medication Reconciliation. http://bjgp.org/content/are-we-legitimately-stopping-medications-use-pharmacist-and-junior-doctor-teaching-improve.

[7] Yeung E.Y.H. (2015). Adverse drug reactions: A potential role for pharmacists. Br. J. Gen. Pract..

[8] Yeung E.Y.H. Collaborative Interprofessional Healthcare Education. http://bjgp.org/content/collaborative-interprofessional-healthcare-education.

[9] Mohammed R.S.D., Yeung E.Y.H. (2017). Physical examinations by pharmacists: Practising the right thing makes perfect. Can. J. Hosp. Pharm..

[10] Yeung E.Y., Comben E., McGarry C., Warrington R. (2015). STI testing in emergency contraceptive consultations. Br. J. Gen. Pract..

[11] Yeung E.Y.H., Chun S., Douglass A., Lau T.E. (2017). Effect of atypical antipsychotics on body weight in geriatric psychiatric inpatients. SAGE Open Med..

[12] Yeung E.Y.H., Buhagiar K. (2017). Correlation of age and metabolic adverse effects of antipsychotics. Clin. Drug Investig..

[13] Lynes S. (2017). Multidisciplinary care. Br. J. Gen. Pract..

[14] Avery A.J. (2017). Pharmacists working in general practice: Can they help tackle the current workload crisis?. Br. J. Gen. Pract..

[15] Yeung E.Y.H. (2017). Explaining the role of pharmacists in multidisciplinary care. Br. J. Gen. Pract..

[16] Yeung E.Y.H. (2017). Pharmacists are not physician assistants. Br. J. Gen. Pract..

[17] McCartney M. (2017). Margaret McCartney: Why GPs are always running late. BMJ.

[18] Irving G., Holden J. (2012). Calling time on the 10-minute consultation. Br. J. Gen. Pract..

[19] Malhotra A., Maughan D., Ansell J., Lehman R., Henderson A., Gray M., Stephenson T., Bailey S. (2015). Choosing Wisely in the UK: The Academy of Medical Royal Colleges’ initiative to reduce the harms of too much medicine. BMJ.

[20] Tomlinson J. (2015). Beyond patient reassurance. Br. J. Gen. Pract..

[21] Kelly D.V., Bishop L., Young S., Hawboldt J., Phillips L., Keough T.M. (2013). Pharmacist and physician views on collaborative practice: Findings from the community pharmaceutical care project. Can. Pharm. J..

[22] The University of British Columbia Interprofessional Health & Human Service Electives. https://socialwork.ubc.ca/current-students/bsw-undergrad-program-info/ihhs-courses/.

[23] Van Winkle L.J., Bjork B.C., Chandar N., Cornell S., Fjortoft N., Green J.M., La Salle S., Lynch S.M., Viselli S.M., Burdick P. (2012). Interprofessional workshop to improve mutual understanding between pharmacy and medical students. Am. J. Pharm. Educ..

[24] Gregory P.A., Austin Z. (2016). Trust in interprofessional collaboration: Perspectives of pharmacists and physicians. Can. Pharm. J..

[25] Abbott A. (1981). Status and status strain in the professions. Am. J. Sociol..

[26] Butterworth J., Sansom A., Sims L., Healey M., Kingsland E., Campbell J. (2017). Pharmacists’ perceptions of their emerging general practice roles in UK primary care: A qualitative interview study. Br. J. Gen. Pract..

